# Differential metabolic effects of constant moderate versus high intensity interval training in high‐fat fed mice: possible role of muscle adiponectin

**DOI:** 10.14814/phy2.13599

**Published:** 2018-02-14

**Authors:** Sergio F. Martinez‐Huenchullan, Babu Raja Maharjan, Paul F. Williams, Charmaine S. Tam, Susan V. Mclennan, Stephen M. Twigg

**Affiliations:** ^1^ Greg Brown Diabetes & Endocrinology Laboratory Sydney Medical School University of Sydney Sydney Australia; ^2^ Faculty of Medicine School of Physical Therapy Universidad Austral de Chile Valdivia Chile; ^3^ Northern Clinical School and Centre for Translational Data Science University of Sydney Sydney Australia; ^4^ New South Wales Health Pathology Sydney Australia; ^5^ Department of Endocrinology Royal Prince Alfred Hospital Sydney Australia

**Keywords:** Adiponectin, exercise, high‐fat diet, skeletal muscle

## Abstract

Exercise regimens may have differing effects in the presence of obesity. In addition to being fat derived, adiponectin has recently been described as a myokine that regulates insulin sensitivity, which may link to exercise‐related metabolic benefits in obesity. Whether skeletal muscle adiponectin varies in different exercise modalities is unclear. This study investigated the comparative effects of 10 weeks of endurance constant‐moderate intensity exercise (END) with high intensity interval training (HIIT), on metabolic outcomes, including muscle adiponectin in a mouse model of diet‐induced obesity. Ten‐week‐old male C57BL/6 mice were fed a high‐fat diet (HFD) (45% FAT) or standard CHOW diet ab libitum and underwent one of three training regimes: (1) no exercise, (2) END, or (3) HIIT (8 bouts of 2.5 min with eight periods of rest of 2.5 min) for 10 weeks (3 × 40 min sessions/week). Chow‐fed mice acted as controls. Compared with HFD alone, both training programs similarly protected against body weight gain (HFD = 45 ± 2; END = 37 ± 2; HIIT = 36 ± 2 g), preserved lean/fat tissue mass ratio (HFD = 0.64 ± 0.09; END = 0.34 ± 0.13; HIIT = 0.33 ± 0.13), and improved blood glucose excursion during an insulin tolerance test (HFD = 411 ± 54; END = 350 ± 57; HIIT = 320 ± 66 arbitrary units [AU]). Alterations in fasting glycemia, insulinemia, and AST/ALT ratios were prevented only by END. END, but not HIIT increased skeletal muscle adiponectin mRNA (14‐fold; *P* < 0.05) and increased protein content of high molecular weight (HMW) adiponectin (3.3‐fold), whereas HIIT induced a milder increase (2.4‐fold). Compared with HFD, neither END nor HIIT altered circulating low (LMW) or high (HMW) molecular weight adiponectin forms. Furthermore, only END prevented the HFD downregulation of PGC1*α* (*P *<* *0.05) mRNA levels downstream of muscle adiponectin. These data show that different training programs affect muscle adiponectin to differing degrees. Together these results suggest that END is a more effective regimen to prevent HFD‐induced metabolic disturbances in mice.

## Introduction

Obesity is related to the development of metabolic dysfunctions (Armani et al. 2017), which has been described in liver (Ban et al. 2016), heart (Hamzeh et al. 2017), and kidney (Whaley‐Connell and Sowers 2017), collectively increasing the risk of cardiovascular disease and mortality. However, particularly in the recent decade, skeletal muscle dysfunction has been increasingly recognized as another detrimental effect of obesity and is characterized by a decrease in muscle strength and reduced ability to endure fatigue (Jurca et al. 2004; Tomlinson et al. 2014; Rastelli et al. 2015). Exercise has been broadly recommended as an effective therapeutic strategy to prevent many obesity‐related metabolic disturbances (Arena et al. 2017). In this context, high‐intensity interval training (HIIT) has arisen as an effective method to improve body composition in overweight and obese adults (Wewege et al. 2017) where the described advantages over more classic regimens (constant‐moderate intensity) are time efficiency and enjoyability (Gibala et al. 2012). Nevertheless, it has been recognized that constant‐moderate intensity exercise programs may have specific benefits that HIIT does not confer (Laursen 2010), such as increases in physical performance at lactate threshold levels (Ingham et al. 2008). However, to our knowledge there are no published studies that have aimed to compare the specific metabolic responses of these two types of exercise, in a high‐fat diet (HFD) context.

One of the mediators of the exercise benefits described in obesity is adiponectin, a 30 kDa protein with a carboxyl‐terminal globular domain and an amino‐terminal collagen domain, enabling it to form multimers (Pajvani et al. 2003; Waki et al. 2003). These multimers are identified by molecular weight as the low‐molecular weight (LMW) and the high‐molecular weight (HMW) isoforms (Kadowaki et al. 2006), with the latter being the most bioactive (Waki et al. 2003). The metabolic relevance of adiponectin resides in its multiple described functions as an insulin‐sensitizer, as well as its anti‐inflammatory and antioxidant activities (Kadowaki et al. 2006). In this context, most of the studies addressing adiponectin have focused on adipose‐tissue secreted adiponectin that can be found in the circulation (Kadowaki et al. 2006). However, adiponectin is also produced by muscle cells and it can act in an autocrine/paracrine manner (Krause et al. 2008; Jortay et al. 2010, 2012). This local muscle bioactivity has been described under various conditions, where mechanical and metabolic stressors were the reported stimuli (Liu et al. 2009; Goto et al. 2013; Pedersen 2013). Increased production of muscle adiponectin has also been described after lipopolysaccharide (LPS) injection (Jortay et al. 2010), exposure to inflammatory cytokines (Delaigle et al. 2004), and HFD (Jortay et al. 2012). Considering these findings, muscle adiponectin production has been proposed as a protective mechanism to counteract the detrimental effects derived from metabolic stressors (inflammation, oxidative stress) (Jortay et al. 2012). Interestingly, for the treatment of metabolic dysfunctions, muscle adiponectin production has been ascribed as one of the beneficial effects of lifestyle interventions (caloric restriction and exercise) (Dai et al. 2013) and it does not appear to be related to change in the level of circulating adiponectin (Garekani et al. 2011). In this context, little is known in obesity about the beneficial effects of different exercise regimens on the muscle adiponectin response. To date, a single study has addressed this question, where after being fed with HFD for 10 weeks, mice showed decreased circulatory HMW adiponectin (Pierard et al. 2016). Interestingly, these changes were partially prevented in a subset of animals that performed 8 weeks of exercise training simultaneously with the HFD intake. The effectiveness of differing exercise programs in inducing this response, particularly related to muscle adiponectin, has not been examined, even though it is well recognized that different exercise regimens induce differential responses in skeletal muscle (Young et al. 2001).

The aim of this study was thus to investigate the effect of 10 weeks of two isocaloric training programs: moderate‐intensity endurance (END) or HIIT, on the expression of muscle adiponectin isoforms and related metabolic outcomes, in a mouse model of diet‐induced obesity. We hypothesized that HFD would change muscle adiponectin isoform content and that both exercise programs, since they were isocaloric, would prevent these changes.

## Materials and Methods

The study was approved by the University of Sydney Animal Ethics Committee (Protocol #2015/816). The experiments described herein were carried out according to the guidelines laid down by the New South Wales Animal Research Act and the eighth Edition of the Australian code for the care and use of animals for scientific purposes.

### Animal characteristics

Seventy‐two C57BL/6 10‐week old male mice were used in this study (Animal Resources Centre, Perth, Australia). Mice were randomly divided into two dietary groups (n = 36/group) and fed either a HFD (45% fat) (prepared in‐house as per [Kim et al. 2015]), or standard laboratory chow (12% fat) (Meat free mouse diet; Specialty Feeds ^®^, WA, Australia), ad libitum, for 10 weeks. Simultaneously, mice in each dietary group were then randomized to one of three training groups: no exercise control group (untrained), constant moderate‐intensity END or HIIT. Consequently, six groups (n = 12/group) were analyzed during this study: CHOW untrained, CHOW + END, CHOW + HIIT; and HFD untrained, HFD + END, HFD + HIIT. Dietary and exercise interventions were delivered simultaneously for 10 weeks and phenotyping studies were performed before and after the intervention. For euthanasia, animals were placed in a sealed box (Stinger ^®^, Advanced Anaesthesia Specialist, Sydney, Australia) and isoflurane (3%) in oxygen was delivered to induce a deep anesthetic state. Then exsanguination through cardiac puncture was performed to euthanize the animals. Blood, quadriceps muscle, subcutaneous, and epidydimal fat depots, were collected and appropriately stored for later analysis.

### Exercise training

After 1 week of treadmill acclimation (6 m/min for 10 min), a maximal running capacity (MRC) test was performed. This progressive test started from a speed of 6 m/min and every three min was increased 3 m/min until exhaustion, defined as the inability of the animal to reach the end of the lane after encouragement with five mechanical stimuli (soft brush) delivered within one minute. The final speed was defined as 100% of the MRC and the distance covered was the aerobic performance of the animal. The exercise intensity of the two different training programs was calculated using the initial MRC. The exercise programs were designed to be isocaloric to rule out differences in energy expenditure from the comparison analysis. This was achieved as follows: for END a running session at 70% of the MRC (17 m/min) for 40 min, and for HIIT eight bouts (2.5 min each) at 90% of the MRC (22 m/min) intercalated by eight active rest periods (2.5 min each) at 50% of the MRC (12 m/min) (40 min total per session). Each training program was performed in the morning, three times per week for 10 weeks. Untrained animals were not exposed to additional exercise of any kind.

### Animal phenotyping

Body weight (g) was measured weekly. Phenotyping experiments were performed 72 h after the last exercise session and consisted of measurement of fat and lean mass by EchoMRI (EchoMRI™ 900 system, Houston, TX) and insulin sensitivity using an insulin tolerance test (ITT) as previously described (Lo et al. 2011). Briefly, during the afternoon after 4 h of fasting, we performed a small nick in the tail with a scalpel and blood (~2 *μ*L per measurement) was obtained from the tail vein of each mouse and blood glucose measured with a specific monitoring system (FreeStyle Lite, Abbott Diabetes Care, Alameda, CA). This measurement was considered as the baseline value. Subsequently, 0.75 IU/kg of body weight intraperitoneal injection of insulin was delivered to each mouse with blood glucose measured at 5, 15, 30, and 60 minutes after insulin injection (Martinez‐Huenchullan et al. 2017). Blood glucose excursion was calculated as area under the curve (AUC) where a lesser reduction in blood glucose, thus a higher AUC, after injection is indicative of greater insulin resistance (Lo et al. 2011).

### Muscle function testing and spontaneous physical activity

To characterize the effect of diet and exercise on muscle function in the animals, we selected two noninvasive tests previously validated by our laboratory for their use in high‐fat fed mice (Martinez‐Huenchullan et al. 2017), namely grip strength and the hang wire test. To assess maximal isometric forelimb grip strength, a Chatillon DFIS 2 Force Gauge was used as described (Martinez‐Huenchullan et al. 2017), each test was performed three times with a 60 sec rest between trials. The mean of these three trials was used for the analysis (De Luca 2014; Bonetto et al. 2015). To assess overall submaximal muscle strength and coordination, the hang wire test was applied. The hang wire test involves placing the mouse on a suspended wire (35 cm height) over a soft fall area for a maximum of three min. The number of reaches to either end of the wire was then counted, as well as the number of falls. The test was stopped if the animal fell more than 10 times (Putten 2016). For ethical reasons, this entire test was performed once only (Martinez‐Huenchullan et al. 2017).

Spontaneous physical activity was measured through a Promethion^®^ Metabolic Cage (Sable Systems International, North Las Vegas, NV). Mice were individually placed in the cage for ~48 h period with ad libitum access to water and food. Displacement and voluntary running wheel usage were counted and combined to obtain the total spontaneous physical activity. Data was collected for 36 h after a 4 h period of acclimatization.

### Plasma insulin, AST/ALT and muscle and plasma triglycerides

Plasma insulin was assessed using a commercial kit (Merck^®^, catalog number EZRMI‐13K and alanine transaminase (ALT) and aspartate transaminase (AST) was measured in the Clinical Chemistry Laboratory of Royal Prince Alfred Hospital using a Roche^®^ (Basel, Switzerland) Cobas auto‐analyser. Triglycerides (TAG) in plasma were quantified using a commercial kit (catalog number: TR0100; Sigma^®^, St. Louis, MO, USA) following kit instructions of the manufacturer. To quantify muscle TAG content ~10 mg of muscle were homogenized in 1000 *μ*L of chloroform:methanol (2:1) extraction reagent. After incubation for 2 h at room temperature, 1 mL of NaCl (0.9% *w/v*) was added and the samples were mixed. To separate the TAG layer, each tube was centrifuged at 1600 *g* for 3 min at room temperature. Next, 200 *μ*L of the infranatant was transferred to a glass tube and dried under a nitrogen stream in a sample concentrator. The resulting pellet was reconstituted with 10 *μ*L of 100% EtOH plus 300 *μ*L of TAG reagent (catalog number 11876023; Roche). After 15 min of incubation at room temperature, absorbance was measured at 492 nm. The muscle TAG content was calculated according to a standard curve and expressed as mg/mL per mg of tissue.

### RNA extraction, reverse transcription, and real time‐qPCR

Real time‐qPCR to determine tissue mRNA levels was performed using standard methods (Martinez‐Huenchullan et al. 2017). In brief, total RNA was extracted from muscle (50‐60 mg) or adipose tissue (150 mg) with TRI reagent (catalog number T9424; Sigma^®^). RNA quantitation and quality assessment were measured with a spectrophotometer (Thermo Fisher Scientific, Nanodrop ^®^, Walthan, MA) and considered acceptable if the 260/230 ratio was above 1.8 in all samples. Reverse transcriptase was performed using 1000 ng of RNA, plus 0.5 *μ*L of oligo(dT) (catalog number 18418‐012; Life Technologies, Carlsbad, CA, USA) at 100 *μ*mol/L, 0.5 *μ*L of random hexamers (catalog number N8080127; Applied Biosystems (Foster City, CA, USA), Life Technologies) at 2 *μ*mol/L, and were incubated at 65°C for 5 min. Then, 4.0 *μ*L of 5× first strand buffer (catalog number 18080‐093; Life Technologies), 0.5 *μ*L of Superscript III reverse transcriptase at 200 U/*μ*L (catalog number 18080‐093; Life Technologies), 2.0 *μ*L of DTT at 0.1M (catalog number 18080‐093; Life Technologies), and 1.0 *μ*L of dNTP mix each at 10 nmol/L (, catalog number BIO‐39025) were added and further incubated at 25°C for 10 min, 50°C for 60 min, and at 70°C for 15 min. Real time‐qPCR was performed on 5 *μ*L of cDNA added to 7.5 *μ*L of SensiMix SYBR Green (catalog number QT605‐20; Bioline, London, UK), 0.5 *μ*L of each primer (forward and reverse) at 10 *μ*mol/L, and 1.5 *μ*L of ddH_2_O. Using a thermocycler (Rotor‐gene Q; QIAGEN ^®^, Hilden, Germany) real time‐qPCR was conducted using a 3‐step procedure as follows: 95°C for 10 min, then 40 cycles of 10 sec at 95°C, 15 sec at 60°C, and 20 sec at 72°C were performed. In each run, a no‐template control was included as a negative control, and after each run a melt curve was performed to confirm the specificity of our in‐house designed primers. Results are expressed using the delta‐delta Ct method, correcting values by the housekeeper gene Rpl7L1 for skeletal muscle as previously published (Thomas et al. 2014) and NoNo for adipose tissue. A list of primer sequences is given in Table 1 for the following genes: Mcp‐1, Adiponectin, AdipoR1, Sirtuin 1, Pgc‐1ɑ, Ucp2, and for the housekeepers, Rpl7L1 and NoNo.

**Table 1:** Primers used for rt‐qPCR

Gene	Forward	Reverse
Mcp‐1	5′‐CACTCACCTGCTGCTACTCA‐3′	5′‐GCTTGGTGACAAAAACTACAG‐3′
Adiponectin	5′‐CGACACCAAAAGGGCTCAGG‐3′	5′‐ACGTCATCTTCGGCATGACT‐3′
AdipoR1	5′‐GCAGACAAGAGCAGGAGTGT‐3′	5′‐TTGACAAAGCCCTCAGCGAT‐3′
Sirtuin 1	5′‐AGCGGCTTGAGGGTAATCAA‐3′	5′‐GAGTATACCTCAGCACCGTGG‐3′
Pgc‐1ɑ	5′‐CTGCGGGATGATGGAGACAG‐3′	5′‐TCGTTCGACCTGCGTAAAGT‐3′
Ucp2	5′‐GGCCTCTGGAAAGGGACTTCT‐3′	5′‐TTGGCTTTCAGGAGAGTATCTTT‐3′
Rpl7L1	5′‐ACGGTGGAGCCTTATGTGAC‐3′	5′‐TCCGTCAGAGGGACTGTCTT‐3′
NoNo	5′‐TGCTCCTGTGCCACCTGGTACTC‐3′	5′‐CCGGAGCTGGACGGTTGAATGC‐3′

### Protein analysis by immunoblotting (Western blot)

Protein expression of adiponectin and ADIPOR1 were determined in muscle and in plasma by Western immunoblot, using established methods (Tam et al. 2015). In detail, for the measurement in muscle, 25‐30 mg of *quadriceps* muscle was homogenized in ice‐cold buffer containing 50 nmol/L Tris HCl, 150 nmol/L NaCl, 1% Triton X‐100, 0.5% Na‐deoxycholate, and 0.1% SDS. After 2 h of incubation at 4°C, samples were centrifuged at 10,000 *g* for 10 min at 4°C. The supernatant was removed and the protein concentration was quantified using a detergent compatible (DC™) protein assay (catalog number 500‐113 and 500‐114; Bio‐Rad^®^, Hercules, CA). Then, 40 *μ*g of protein was loaded to a precast polyacrylamide gradient gel (4–15%) (catalog number 4568086; Bio‐Rad^®^). For the studies in plasma, 0.5 *μ*L of sample was mixed with 3.8 *μ*L of loading buffer and 10.7 *μ*L of RIPA buffer and loaded directly onto the gels. The proteins contained within the gel were transferred to a nitrocellulose membrane (catalog number 1704158; Bio‐Rad^®^) using the Trans‐Blot^®^ Turbo™ Transfer System (Bio‐Rad). Membranes were blocked for 1 h using 5% skim milk in buffer (TBST) containing 0.6% Tris HCl *w/v*, 0.1% Tris‐base *w/v*, 0.6% NaCl *w/v*, and 0.05% Tween‐20 *v/v*. Membranes were then washed for 10 min three times with TBST and incubated in primary antibodies as follows and as per the manufacturer instructions: Adiponectin 1:2000 (rabbit polyclonal GeneTex [Irvine, CA, USA], catalog number GTX23455), Adiponectin receptor 1 (ADIPOR1) 1:2000 (rabbit polyclonal GeneTex, catalog number GTX32425) overnight at 4°C. Membranes were washed as before in again in TBST, and then incubated with a secondary antibody labelled with peroxidase (1:10,000, Anti‐rabbit IgG, catalog number S9169; Sigma^®^) for 1 h at room temperature. Membranes were then washed with TBST and developed with a chemiluminescent substrate (Clarity™ Western ECL substrate, Bio‐Rad^®^, catalog number 170‐5061) and visualized on a Chemidoc imaging system (Bio‐Rad^®^). Densitometric analysis of the bands was performed using Image Lab software (Bio‐Rad^®^). Protein loading was confirmed and normalized using Ponceau S staining of the whole membrane in each respective experiment.

### Histology and immunohistochemistry

Immunohistochemistry to detect adiponectin was also undertaken using standard methods (Martinez‐Huenchullan et al. 2017). In detail, samples were fixed in 10% formalin for 12 h then changed to 80% EtOH (*v/v*) for overnight processing. From the paraffin‐embedded blocks, 5 *μ*mol/L sections were stained with hematoxylin‐eosin to investigate the muscle global structure. Adiponectin staining was achieved by immunohistochemistry, with antigen retrieval (microwave at pH 6.0 citrate buffer for 30 min). After cooling antigenic sites were incubated in 3% H_2_O_2_ for 5 min and then 1% BSA in TBST for 30 min, prior to overnight incubation at 4°C in the primary antibody against adiponectin 1:500 (rabbit polyclonal GeneTex, catalog number GTX23455). After secondary antibody incubation for 30 min (1:200, Biotinylated anti‐rabbit IgG, catalog number BA‐1000; Vector Laboratories^®^, Burlingame, CA, USA) at room temperature, slides were incubated in avidin‐biotin complex (Vectastain ABC kit, catalog number PK‐4000; Vector Laboratories^®^) and diaminobenzidine (DAB) (catalog number K3468; Dako^®^, Santa Clara, CA, USA) was added to obtain a detectable brown color. The reaction was terminated by washing the slides in ddH_2_O. Slides were seen and pictures taken with an Olympus BX53 microscope, with cellSens microimaging software (Olympus, Tokyo, Japan).

### Statistical analysis

Statistical analyses were performed using GraphPad software Version 7.0. Normally distributed data were expressed as mean ± SD. Non‐normally distributed data were expressed as median (interquartile range). Effects of dietary interventions (CHOW vs. HFD) and the differences between exercise conditions (untrained vs. END vs. HIIT) were calculated using a Two‐way ANOVA with Dunnet's and Sidak's pos‐hoc analysis. *P* values <0.05 were considered statistically significant.

## Results

### Diet and training effects on animals phenotype

**Figure 1:**
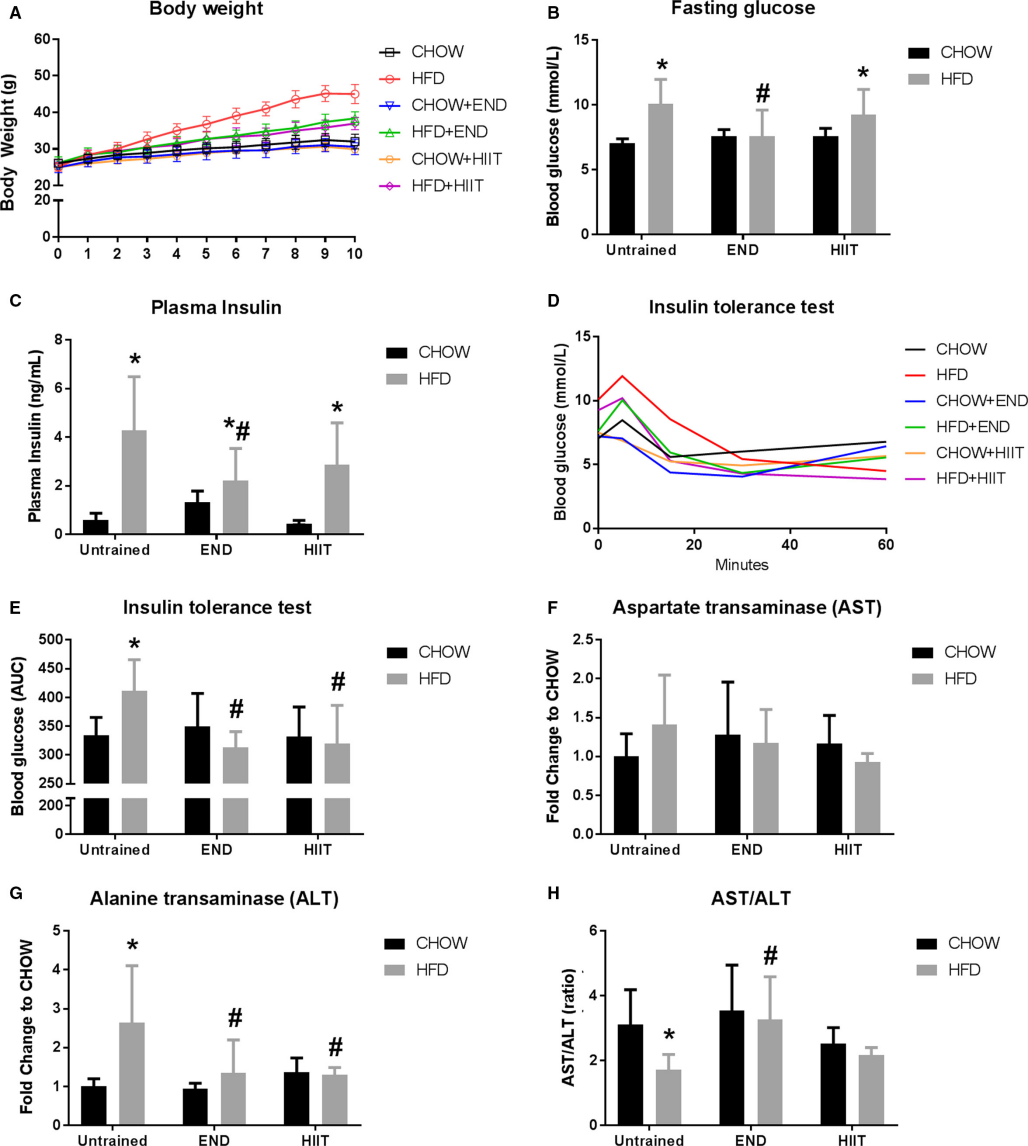
Body weight and metabolic markers after exercise training. Weekly changes in body weight (A), fasting glucose (B), plasma insulin (C), blood glucose excursion (D), and area under the curve (AUC) (E) after the insulin tolerance test, plasma aspartate transaminase (AST) (F), plasma alanine transaminase (ALT) (G), and the ratio between the circulatory levels of AST and ALT (H). Data are presented as mean ± SD and the number of animals per group is 8–12. *P <* *0.05 versus CHOW untrained; #*P *<* *0.05 versus HFD untrained by two‐way ANOVA with Dunnet's and Sidak's post‐hoc tests.

Most of the animals in the exercise groups completed the training program. The groups CHOW + END, CHOW + HIIT, and HFD + END had one drop‐out each whereas HFD + HIIT had four. For all of them, the reason was lack of compliance in the training sessions. Because of this, these mice were excluded from this experiment and were included in other parallel projects of our group. The final number of mice per group were: CHOW untrained = 12, CHOW + END = 11, CHOW + HIIT = 11, HFD untrained = 12, HFD + END = 11, HFD + HIIT = 8. Phenotypic changes after 10 weeks of HFD and exercise training are presented in Table 2. As expected, HFD induced an increase in body weight (Fig. 1A), which was partially attenuated by END and by HIIT training (both *P *<* *0.05). These changes coincided with the partial restoration of the proportion between fat and lean mass in high‐fat fed animals that were exercised. Interestingly, hyperglycemia and hyperinsulinemia driven by HFD were only prevented after END (Fig. 1B and C). Similarly, dysregulations in the plasma AST/ALT ratio were prevented only after END (Fig. 1H) with no changes in AST (Fig. 1F) and a similar preventive effect in ALT plasma levels (Fig. 1G). However, when mice were challenged during the ITT, END, and HIIT showed a comparable effect in preventing the increase in the blood glucose excursion (Fig. 1D–E), indicating a more insulin responsive state was achieved with both exercise regimens in HFD mice.

**Table 2:** Animal characteristics at termination

Parameter	CHOW			HFD		
	Untrained (n = 12)	END (n = 11)	HIIT (n = 11)	Untrained (n = 12)	END (n = 11)	HIIT (n = 8)
Body weight (g)	32.5 ± 1.7	31.0 ± 1.8	30.7 ± 1.2	45.2 ± 2.2*	37.4 ± 2.1* ^,^ #	35.9 ± 2.0* ^,^ #
Fat/lean mass (ratio)	0.14 ± 0.05	0.11 ± 0.03	0.09 ± 0.03	0.64 ± 0.09*	0.34 ± 0.13* ^,^ #	0.33 ± 0.13* ^,^ #
Quadriceps weight (g)	0.24 ± 0.02	0.22 ± 0.04	0.23 ± 0.01	0.28 ± 0.05*	0.21 ± 0.03#	0.22 ± 0.03#
Subcutaneous Adipose Tissue (%BW)	0.9 ± 0.3	0.9 ± 0.2	0.8 ± 0.1	2.9 ± 0.5*	1.8 ± 0.5* ^,^ #	1.8 ± 0.6* ^,^ #
Epidydimal Adipose Tissue (%BW)	2.1 ± 0.6	1.9 ± 0.4	1.7 ± 0.4	5.8 ± 0.9*	4.6 ± 1.1* ^,^ #	4.9 ± 1.3* ^,^ #
Spontaneous physical activity (AU)	316 ± 75	354 ± 51	322 ± 87	211 ± 59*	308 ± 79#	304 ± 77#

Data are expressed as mean ± SD. AU, arbitrary units; %BW, percentage of body weight; HIIT, high intensity interval training; END, endurance; HFD, high‐fat diet.

*P <* *0.05 versus CHOW untrained; #*P *<* *0.05 versus HFD untrained by two‐way ANOVA.

**Figure 2:**
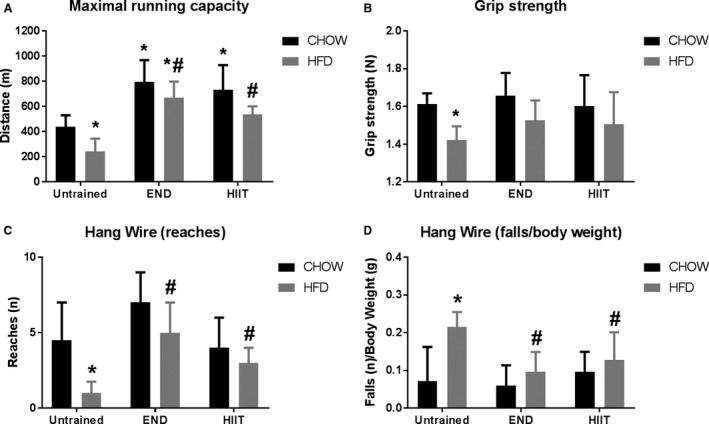
Muscle function after training. Distance covered in the maximal running capacity test after training (A), grip strength (B), number of reaches (C) and falls (normalized by body weight) (D) in the hang wire test. Data are presented as mean ± SD (A, B) and as median ± interquartile range (C, D) and the number of animals per group is 8–12. *P <* *0.05 versus CHOW untrained; #*P *<* *0.05 versus HFD untrained by two‐way ANOVA with Dunnet's and Sidak's post‐hoc tests.

In terms of muscle function, HFD compared with CHOW caused a decrease in maximal (grip strength) and submaximal (decrease in hang wire reaches and increase in falls) muscle strength, which was similarly prevented by END and HIIT (Fig. 2B–D). Along with these changes, a significant decrease in spontaneous physical activity (~33%) was seen in HFD untrained mice which was prevented by END and HIIT in a similar fashion (Table 2). As expected, increases in MRC were seen after exercise training in CHOW and HFD mice (Fig. 2A). Under the standard (CHOW) diet, no differences were seen between exercise programs on the MRC; interestingly, in high‐fat fed mice, the animals that were in the END group performed better than CHOW untrained animals, which was not seen after HIIT.

### Muscle lipid accumulation and TAG

**Figure 3:**
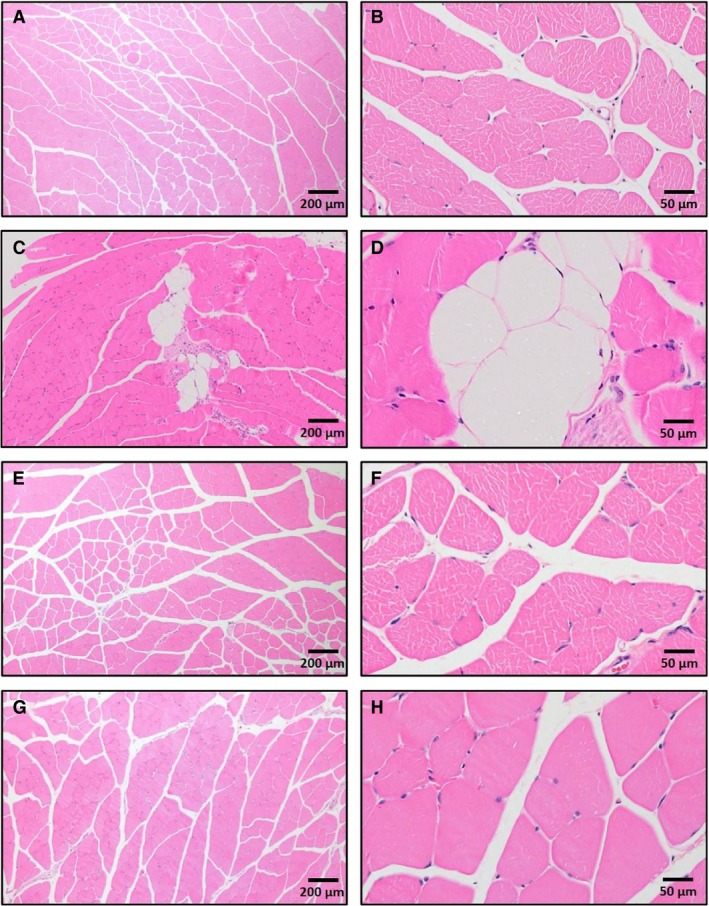
Representative cross‐sectional *quadriceps* muscle sections, with haematoxylin and eosin staining at magnifications for each condition of 4× (left) and 20× (right). CHOW untrained (A, B), high‐fat fed untrained (C, D), high‐fat fed after endurance training (HFD + END; E, F), high‐fat fed after high‐intensity interval training (HFD + HIIT; G, H). The effects of high‐fat diet are clearly shown in C and D with fat infiltration between fascicles. No fat infiltration was seen in muscle tissue from mice in either of the training programs. Since no major differences were seen in CHOW mice after exercise training, those images are not shown.

**Figure 4:**
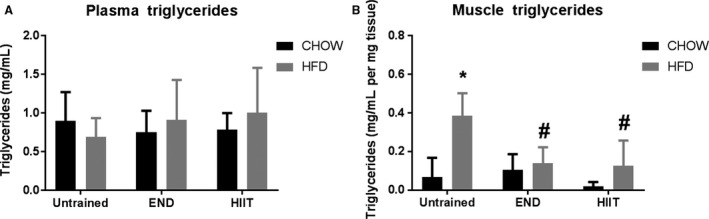
Plasma and muscle tissue triglyceride content. Plasma (A) (n = 8–12 per group) and muscle (B) (n = 5 per group) triglyceride content. Data are presented as mean ± SD. *P <* *0.05 versus CHOW untrained; #*P *<* *0.05 versus HFD untrained by two‐way ANOVA with Dunnet's and Sidak's post‐hoc tests.

Fat accumulation in muscle was assessed using hematoxylin & eosin staining, as shown in Figure 3. Signs of fat infiltration found in HFD untrained mice were prevented by END and HIIT without obvious differences between exercise programs. In support of this, the levels of circulating TAG in plasma were not changed after HFD and/or exercise (Fig. 4A), whereas the expected increase in muscle TAG driven by HFD, was similarly prevented by END and by HIIT (Fig. 4B).

### Adiponectin and associated factors after HFD and exercise in muscle and adipose tissue

**Figure 5:**
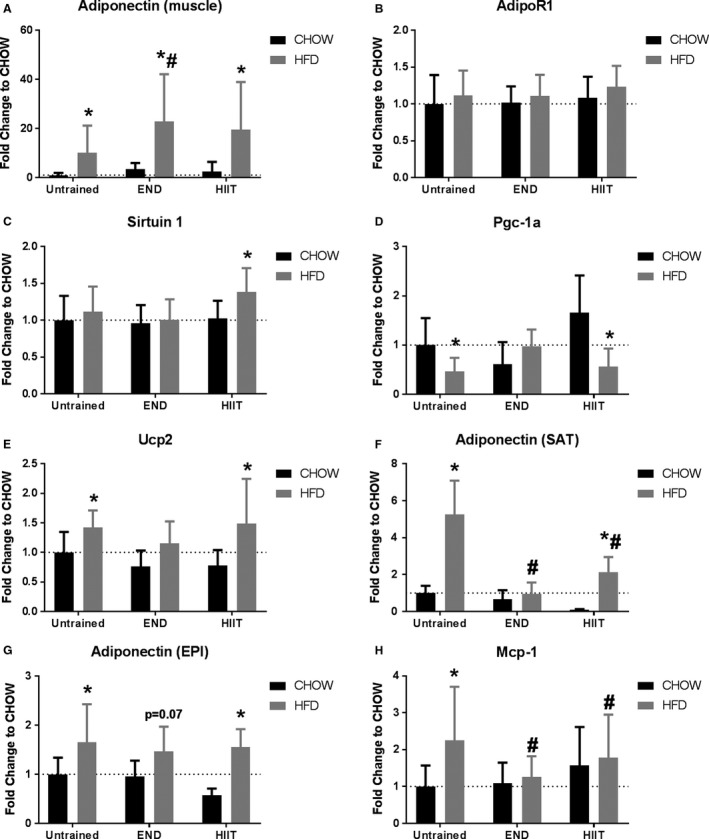
Adiponectin steady state mRNA levels in muscle and adipose tissue. mRNA levels of muscle adiponectin (A), adiponectin receptor 1 (AdipoR1, B), downstream molecules sirtuin 1 (C), peroxisome proliferator‐activated receptor gamma coactivator 1‐alpha (Pgc‐1ɑ, D), uncoupling protein 2 (Ucp2, E), subcutaneous (SAT) adiponectin (F), epidydimal (EPI) adiponectin (G), and the inflammation mediator monocyte chemotactic protein‐1 (Mcp‐1, H). High‐fat diet (HFD), endurance training (END), high‐intensity interval training (HIIT). Data are presented as means ± SD and the number of animals per group is 8–12. *P <* *0.05 versus CHOW untrained; #*P *<* *0.05 versus HFD untrained by two‐way ANOVA with Dunnet's and Sidak's post‐hoc tests.

To examine the potential mechanism by which exercise induced its metabolic effects in skeletal muscle, adiponectin levels were determined. HFD induced an up‐regulation of muscle adiponectin mRNA levels in untrained mice (~10‐fold) (Fig. 5A); interestingly, END induced a further increase (more than 20‐fold). This response was similar after HIIT, however, it did not reach statistical difference compared with HFD untrained mice. To confirm these changes, mRNA of Adiponectin receptor 1 (AdipoR1) and three factors known to be regulated downstream of adiponectin were then measured in muscle. No effect of diet or exercise was seen in AdipoR1 (Fig. 5B). No diet effect in untrained animals was seen in Sirtuin 1, but a mild increase (1.3‐fold) in HFD mice that underwent HIIT (Fig. 5C). Down‐ regulation of peroxisome proliferator‐activated receptor gamma coactivator 1‐alpha mRNA (Pgc‐1ɑ) seen after the HFD was only prevented by END (Fig. 5D). Similar results were seen where uncoupling protein 2 (UCP2) mRNA levels were increased by HFD (~1.5‐fold) and this was prevented only by END (Fig. 5E). HFD promoted a significant increase in adiponectin mRNA levels in subcutaneous adipose tissue (~fivefold) (Fig. 5F). This increase was prevented by END and only partially by HIIT. In epidydimal adipose tissue a mild upregulation was seen (~1.5‐fold) in all HFD groups, that did not reach statistical significance after END (Fig. 5G). To investigate if HFD promoted an inflammatory state in muscle the mRNA levels of monocyte chemotactic protein‐1 (Mcp‐1, a known inflammatory marker in muscle [Patsouris et al. 2014]) was determined, and was found to be upregulated (~twofold) in HFD untrained animals, whereas END and HIIT similarly prevented this change (Fig. 5H).

**Figure 6:**
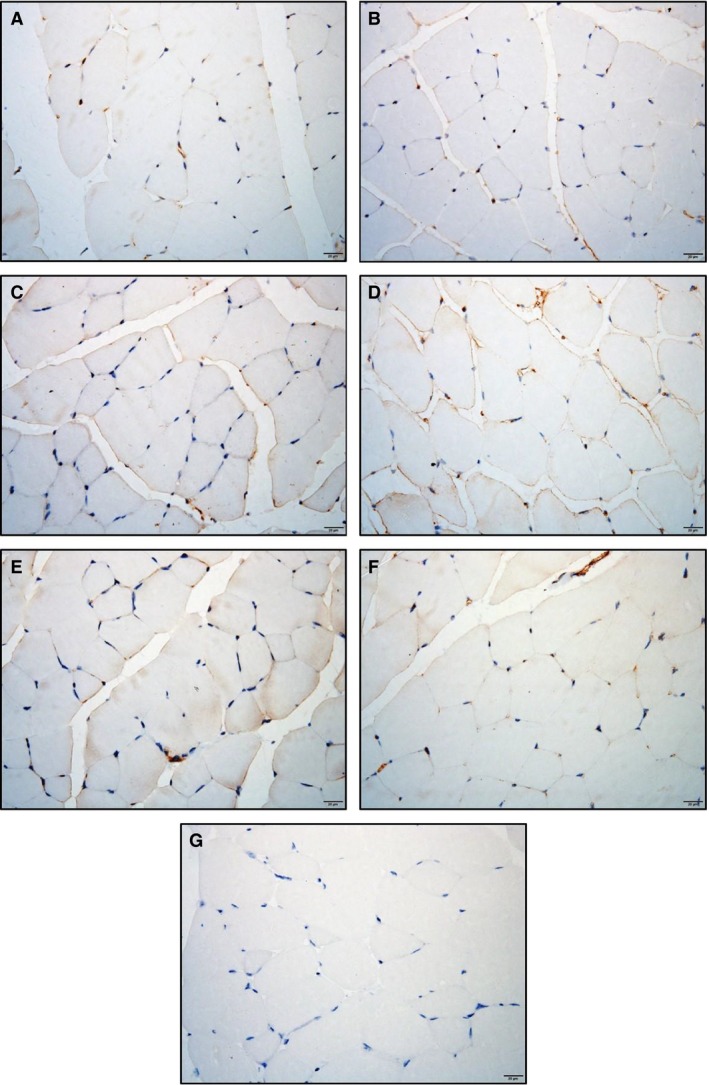
Localization of muscle adiponectin. Representative cross‐sections of *quadriceps* muscle with adiponectin immunohistochemistry determined by immunoperoxidase stain,and nuclear counter‐staining with haematoxylin. CHOW untrained (A), high‐fat diet untrained (B), CHOW + END (C), HFD + END (D), CHOW + HIIT (E), and HFD + HIIT (F) are displayed. No striking differences were seen across the different groups. Positive staining was mainly found in the sarcolemma followed by a less intense signal in the cytoplasmic region. A negative control without primary antibody is shown in (G). The size bars indicate 20 *μ*m and images are at 40× magnification.

**Figure 7:**
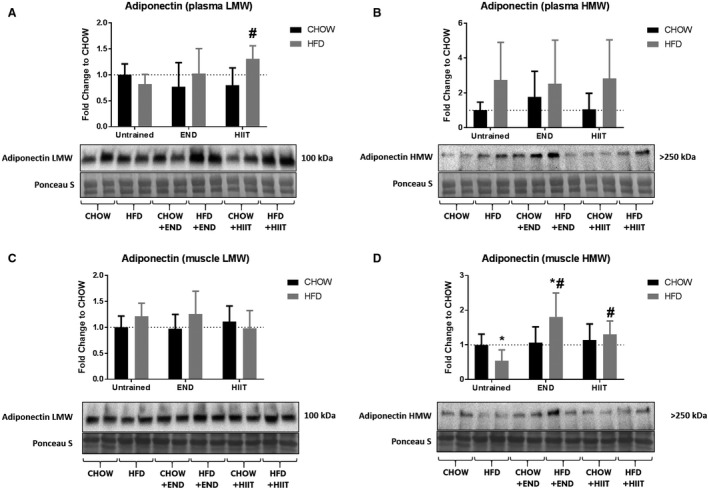
Adiponectin isoforms in plasma and muscle. Plasma low‐molecular weight (LMW) adiponectin (A) and high‐molecular weight (HMW) adiponectin (B); muscle LMW (C) and HMW (D), and their representative blots below its respective graph. Data are presented as means ± SD and the number of animals in each group is 8. *P <* *0.05 versus CHOW untrained; #*P *<* *0.05 versus HFD untrained by two‐way ANOVA with Dunnet's and Sidak's post‐hoc tests.

Considering that skeletal muscle adiponectin mRNA was found to be regulated by HFD and impacted upon by exercise, adiponectin protein profiling in *quadriceps* muscle was then determined by IHC, as shown in Figure 6. A clear extracellular staining accompanied by a less intense cytoplasmic staining was observed, particularly in END groups (both CHOW and HFD) (Fig. 6C and D). To assess the differing isoforms in muscle and circulation, the levels of adiponectin were then semiquantitatively determined by western immunoblotting (Fig. 7). In plasma, no major changes were seen after HFD and/or exercise in the HMW adiponectin, and only a mild increase in the LMW (~30%) was detected in HFD + HIIT mice (Fig. 7A and B). In muscle, the LMW was not significantly affected by HFD or exercise (Fig. 7C), whereas most of the changes were seen in the HMW isoform: lower levels of the HMW isoform were detected in HFD untrained mice (Fig. 7D), a process that was prevented by both exercise programs. Interestingly, END promoted a greater upregulation (3.3‐fold vs. HFD untrained) of this isoform compared with HIIT (2.4‐fold vs. HFD untrained) (Fig. 7D).

**Figure 8:**
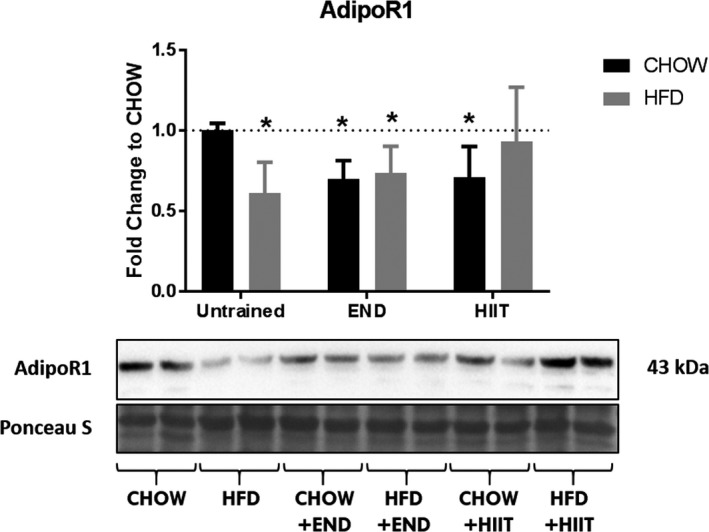
Adiponectin receptor 1 (AdipoR1) content in muscle. AdipoR1 content in *quadriceps* after the experiment, and its representative blot below the graph. *P <* *0.05 versus CHOW untrained. Data are presented as means ± SD and the number of animals in each group is 6. *P <* *0.05 versus CHOW untrained by two‐way ANOVA with Dunnet's and Sidak's post‐hoc tests.

To determine whether HFD and/or exercise affected the levels of adiponectin receptor in muscle, the level of AdipoR1, (the most abundant adiponectin receptor in this tissue [Jortay et al. 2012]), was determined. Lower levels of AdipoR1 were detected after HFD alone and in the HFD group undergoing END (Fig. 8); interestingly, this decrease was found in CHOW + END and in CHOW + HIIT mice as well, but no changes in the levels of AdipoR1 were seen in HFD + HIIT mice.

## Discussion

This is the first study that compares the metabolic effects of constant‐moderate intensity (END) and HIIT, along with the muscle adiponectin response, in the context of diet‐induced obesity. We found that HFD promoted a downregulation of muscle HMW adiponectin protein and that both exercise programs were able to prevent this dysregulation. Furthermore, a differential response between the two exercise programs was observed, where END induced a further increase in muscle HMW adiponectin, a change that was associated with the prevention of hyperglycemia, hyperinsulinemia and dysregulation of the AST/ALT ratio in high‐fat fed animals that underwent this type of training, which was not seen after HIIT exercise training. In this context, the similarity of the exercise programs designed in this study to what is commonly suggested in clinical practice in terms of type, frequency and duration (compared for example with voluntary running wheel usage), highlights the clinical relevance of the different exercise effects utilized in this study.

Obesity is a condition that can disrupt muscle function, decreasing its capacity to produce force and to endure fatigue (Tomlinson et al. 2014; Hasan et al. 2016). In support of this effect, HFD untrained mice showed impaired muscle performance in grip strength and hang wire tests performed in this study. Since both exercise programs were considered aerobic, an increase in the MRC in trained animals was expected. However, prevention of decreases in maximal (grip strength) and submaximal muscle strength (hang wire) are not considered to be main effects of aerobic exercise, where resistance training is classically recommended for these outcomes (Schmitz et al. 2003). Therefore, we think that these changes in muscle strength are related to a preventive effect of exercise, maintaining a healthier muscle structure and metabolic function instead of an exercise‐driven anabolic signaling. Highlighting the relevance of muscle adiponectin in physiology, decreases in peak force have been described in adiponectin‐null mice (Krause et al. 2008). In support of this, in mice after 2 weeks of hindlimb suspension and 2 weeks further of reloading, increases in adiponectin were found in the *soleus* muscles of wildtype mice, which accorded with a normalization of muscle weight otherwise decreased after suspension (Goto et al. 2013). In the same study, similar findings were described after 3 weeks of *soleus* overloading by dissection of agonists (Goto et al. 2013). Moreover, muscle transfection of adiponectin gene in insulin‐resistant rats reversed decreases in peak twitch and tetanic tension in *gastrocnemius*, in a similar fashion to a swimming exercise program (Safwat et al. 2013).

One of the difficulties regarding the studies associated with adiponectin response is that this process seems to be sensitive to variations in models and settings. It is globally accepted that hypoadiponectinemia is one of the consequences of obesity (Kadowaki et al. 2006; Chiarugi and Fiaschi 2010). However, Kuo & Halpern, after collecting data from 43 different groups (~5000 human cohort) failed to find a correlation between circulating concentration of adiponectin and body mass index (BMI), even when adults with a BMI higher than 30 kg/m^2^ were included (Kuo and Halpern 2011). This suggests that obesity may not be the sole factor inducing these changes, whereas metabolic disorders associated with this condition could have a stronger influence. In support of this, and in agreement with our study, no major changes in circulating adiponectin after four (Bonnard et al. 2008), 8 (Keshvari et al. 2017) and 10 weeks (Pierard et al. 2016) of high‐fat feeding in mice have been described by others. In this context, the increase in mRNA levels of adiponectin in the subcutaneous and epidydimal fat depots in the untrained HFD animals could mask any changes in the circulating content of this protein. In contrast, increases in plasma adiponectin were reported in mice after 16 weeks of high fat/high sucrose diet (Bonnard et al. 2008), however, only in the low molecular weight form of adiponectin (30 kDa), highlighting the relevance of measuring the different adiponectin isoforms before any conclusion can be made (Waki et al. 2003).

Contrary to the discrepancy related to circulating levels, evidence regarding muscle‐derived adiponectin is scarcer yet its published findings are concordant. One of the earliest studies addressing this issue from Delaigle et al. (2004) reported that after intramuscular LPS injections in mouse *tibialis anterior*, increases in muscle adiponectin transcription (~10‐fold) and content (~1.5‐fold) were found, a process that was replicated in C_2_C_12_ myotubes after they were cultured in media with interferon‐ɣ + TNF‐ɑ. This response was considered a protective mechanism of skeletal muscle against muscle inflammation. Further validation of this hypothesis was made in global adiponectin KO mice where a similar LPS injection induced more severe signs of damage and degeneration compared with wild‐type controls. Interestingly, this damage was attenuated after the electroporation of adiponectin‐containing plasmids in the affected muscles. Remarkably, these changes were independent of variations in the circulating concentration of adiponectin (Jortay et al. 2010). In our study, to investigate if muscle inflammation was playing a role in this process, we selected MCP‐1 as a marker of inflammation, given its relationship with the development of muscle insulin resistance (Patsouris et al. 2014). We found, as expected, that muscle MCP‐1 mRNA was upregulated in high‐fat fed mice and this increase was prevented by exercise. Nevertheless, more studies are needed in this area to clarify an eventual difference between training regimes regulating the HFD‐driven muscle inflammation.

In the last decade, muscle adiponectin induction has been tested in animal models of obesity, to investigate if metabolic stressors in the form of excess of energy intake could change adiponectin levels. In one study, in *tibialis anterior* muscles of 16‐weeks old *ob/ob* mice, increased transcription and content of adiponectin was found, with values that were partially reversed after treatment with the anti‐hyperlipidemic drug, probucol (Delaigle et al. 2006). However, it seems that the biological function of adiponectin is undermined in an obesity context, where a negative effect of over nutrition has been described on the action of this protein in terms of promoting fat utilization for energy production (Krause et al. 2008; Ritchie and Dyck 2012). Interestingly, Yang et al. (2006) found major decreases (50%) in adiponectin content in rat *gastrocnemius* after 20 weeks of high‐fat/high‐sucrose diet, arising questions about the relevance of the animal model chosen to study these changes. In that context, and accordingly to what Yang et al. described, in our study we found that HFD induced a clear downregulation of HMW muscle adiponectin.

That adiponectin can form multimers in muscle may drive the variability in the findings of different reports. Bonnard et al. (2008) found an increase in muscle adiponectin in mice after 16 weeks of HFD, however, they only measured the monomer form (30 kDa). Similar findings are described when total adiponectin is measured directly in skeletal muscle (by immunohistochemistry) (Delaigle et al. 2006; Jortay et al. 2012). In contrast, and consistent with our findings, decreases in the most bioactive isoform (HMW) were found previously where the effects of HFD on muscle adiponectin were investigated (Liu et al. 2009). This decrease in muscle HMW adiponectin occurring in an obesity context does not yet have a clear mechanistic explanation, however, mutations in the adiponectin gene found in patients with metabolic syndrome (Waki et al. 2003) and signs of obesity‐induced DNA hypermethylation of the adiponectin gene in animal models (Kim et al. 2015) may in the future bring some light to this topic.

Since exercise has multiple metabolic benefits in overweight and obese individuals (Wewege et al. 2017), it is of value to investigate the preclinical effects of exercise, and its differing types, in this context. To our knowledge, just one study has addressed this question and with one exercise type only. Pierard et al. (2016) trained C57BL/6 mice for 10 weeks on a constant moderate‐intensity protocol, similar to our END training, where the major differences were that the intensities and time length of the sessions were increased slightly every week. At the end of the experiment, in agreement with our results, they found improvements in insulin sensitivity (through glucose tolerance tests). Moreover, they also found a prevention of the downregulation of circulatory HMW adiponectin in trained animals. Interestingly, they also reported a total prevention of the downregulation of muscle AdipoR1 in trained HFD mice. Notably, all mice were sacrificed 24 h after the last training session, implying that some of the acute effects of exercise were present in the target tissues, as seen by others in human skeletal muscle (Bickel et al. 2005). These findings plus the downregulation of AdipoR1 that we found 72 h after the last bout of exercise could indicate that variations in the concentrations of this receptor in muscle are dynamic and dependent on the exercising status of muscle (active vs. inactive).

In our study, END was able to prevent the expected hyperglycemia and hyperinsulinemia after HFD. In this context, Hu et al. (2017) in high‐fat fed and streptozotocin‐induced diabetic mice found that different concentrations of intravenous‐delivered adiponectin (10, 25, and 100 mg/kg) protected the animals against hyperglycemia, which may be one of the reason why END was also able to partially protect the animals against hyperinsulinemia. However, no clear mechanisms were explained. Moreover, given that the differences that we found were localized in the skeletal muscle, this point remains to be further investigated. Nevertheless, to test if the changes in the different muscle adiponectin isoforms may be related to significant metabolic effects on its known pathways, we measured three known factors in skeletal muscle that are directly and indirectly regulated by adiponectin, namely Sirtuin 1 (Iwabu et al. 2010), PGC‐1ɑ (Iwabu et al. 2010), and UCP‐2 (Liu and Sweeney 2014). The expected downregulation of PGC‐1ɑ (Sparks et al. 2005) and up‐regulation of UCP‐2 (Schrauwen et al. 2001) by HFD were only prevented by END, suggesting a stronger adiponectin signaling in muscle under this type of training rather than by HIIT. With the outcomes of this study, it is not clear as to how END induced a higher muscle HMW adiponectin upregulation compared with HIIT, however, since END was designed as a moderate‐intensity regimen, it is expected that an aerobic pathway for energy production was stimulated (fat oxidation), thus addressing in a more effective manner the higher fat influx from the diet. On the contrary, HIIT has a mixture of anaerobic and aerobic phases which, in a HFD context, may hinder this adaptation process, given that a mixture of nutrients (carbohydrates and fats) are required for energy production under this kind of regimen (Lieber 2010). We thus hypothesise that muscles under an END regimen were able to produce higher levels of muscle HMW adiponectin related to the preferential fat oxidation induced by END compared with HIIT; more mechanistic studies addressing this hypothesis directly will be required in a separate study. Further experiments should also aim to investigate if there are differences in the levels of AMPK phosphorylation, protein content and activity of Sirtuin 1, and/or changes in the muscle content of ceramides after these different types of exercise, given that in addition to UCP‐2 and PGC‐1ɑ, these factors have also been described as targets of adiponectin action in skeletal muscle (Hoeg et al. 2013; Iwabu et al. 2010). Considering the limitations of the present study, the inclusion of only male mice does not allow us to consider gender specific exercise and metabolic responses as female adiponectin levels are higher in females than in males. Furthermore, that we did not include a resistance training component in the study we are unable to extrapolate these findings to mixed exercise programs (aerobic + resistance).

In conclusion, HFD induces a dysregulation in muscle adiponectin isoforms that is similarly prevented by END and HIIT. Moreover, END seems to induce preferential metabolic benefits characterized by prevention of hyperglycemia, hyperinsulinemia, and improvements in markers of liver damage (AST/ALT), which were paralleled by further increases in muscle HMW adiponectin and normalization of the mRNA levels of direct and indirect downstream effectors of the protein observed after this type of training. Further research should be aimed at clarifying how END has more desirable effects than HIIT on skeletal muscle and in some key systemic metabolic measures, and whether skeletal muscle adiponectin is mediating these effects.

## Conflicts of Interest

There are no conflicts of interest for any of the authors related to this manuscript.
